# Selection of reference genes for normalization of RT-qPCR data in gene expression studies in *Anthonomus eugenii* Cano (Coleoptera: Curculionidae)

**DOI:** 10.1038/s41598-020-61739-z

**Published:** 2020-03-19

**Authors:** Daniele H. Pinheiro, Blair D. Siegfried

**Affiliations:** 1 University of Florida, Entomology and Nematology Department, Charles Steinmetz Hall, P. O. Box 110620, Gainesville, FL 32611–0620 United States; 2 Present Address: Embrapa Genetic Resources and Biotechnology, Parque Estação Biológica, W5 Norte, P. O. Box 02372, Brasília, DF 70770–917 Brazil

**Keywords:** RNAi, RNAi, RNAi, RNA, RNA

## Abstract

The pepper weevil, *Anthonomus eugenii* Cano (Coleoptera: Curculionidae), is the main insect pest of peppers (*Capsicum* spp.) throughout the southern U.S. and a potential target for novel control methods that may require gene expression analyses. Careful selection of adequate reference genes to normalize RT-qPCR data is an important prerequisite for gene expression studies since the expression stability of reference genes can be affected by the experimental conditions leading to biased or erroneous results. The lack of studies on validation of reference genes for RT-qPCR analysis in *A. eugenii* limits the investigation of gene expression, therefore it is needed a systematic selection of suitable reference genes for data normalization. In the present study, three programs (BestKeeper, geNorm and NormFinder) were used to analyze the expression stability of candidate reference genes (*β-ACT*, *ArgK*, *EF1-α*, *GAPDH*, *RPL12*, *RPS23*, α-*TUB*, *18S* and *28S*) in *A. eugenii* under different experimental conditions. Our results revealed that the most stably expressed reference genes in *A. eugenii* varied according to the experimental condition evaluated: developmental stages (*EF1-α*, *18S* and *RPL12*), sex (*RPS23* and *RPL12*), low temperature (*GAPDH* and *α-TUB*), high temperature (*α-TUB* and *RPS23*), all temperatures (*α-TUB* and *GAPDH*), starvation (*RPL12* and *α-TUB*), and dsRNA exposure (*α-TUB* and *RPL12*). Our study provides for the first time valuable information on appropriate reference genes that can be used in the analysis of gene expression by RT-qPCR in biological experiments involving *A. eugenii*.

## Introduction

Reverse-transcription quantitative PCR (RT-qPCR) is widely used in gene expression studies due to its simplicity, reproducibility, high sensitivity, accuracy and cost-effectiveness^1,2^. Although RT-qPCR is considered a highly accurate technique, several experimental factors can lead to results that are not reliable measurements of gene expression. These factors include purity and integrity of RNA, quantity of starting RNA and cDNA, reverse transcription and PCR efficiency, and pipetting errors^3^. Thus, in RT-qPCR analysis is necessary to use reference genes to normalize the data in order to eliminate or at least reduce the technical variation among the tested samples and precisely estimate the expression of the target genes^4^.

Usually, housekeeping genes related to basic cellular functions are used as reference genes in the normalization strategy because these genes are supposed to have constitutive and stable expression under a variety of physiological conditions and experimental treatments. However, several studies have demonstrated that the expression of housekeeping genes is not always stable and can be influenced by developmental stage, tissue, sex, and biotic or abiotic stresses that the organism is subjected^5–9^. Therefore, the selection of suitable reference genes according to the specific experimental conditions is essential to ensure accurate results.

The pepper weevil, *Anthonomus eugenii* Cano (Coleoptera: Curculionidae), is the most economically important pest of cultivated peppers (*Capsicum* spp.) in the southern United States, Mexico, Central America and some Caribbean islands^10–15^. *A. eugenii* larvae feed preferentially inside floral buds and immature fruits, while adults feed on buds, flowers, fruits, and even young leaves. Premature abscission of the fruits as a result of larval and adult feeding leads to losses in the production of marketable fruits which is the main damage caused by this insect^16^.

Gene expression analysis is an important tool to improve the understanding of molecular and genetic processes in *A. eugenii*, which in turn may provide insight into the development of novel management strategies for this emerging insect pest, such as RNAi-based control methods. However, optimal reference genes for RT-qPCR data normalization have not yet been identified in *A. eugenii*, thereby limiting further studies of gene expression. In this study, the stability of nine candidate reference genes, including *β-Actin* (*β-ACT*), *Arginine kinase* (*ArgK*), *Elongation factor 1-α* (*EF1-α*), *Glyceraldehyde-3-phosphate dehydrogenase* (*GAPDH*), *60S ribosomal protein L12* (*RPL12*), *40 ribosomal protein S23* (*RPS23*), *α-Tubulin* (α-*TUB*), *18S ribosomal RNA* (*18S*) and *28S ribosomal RNA* (*28S*) was evaluated using three statistical algorithms, BestKeeper, geNorm and NormFinder. In addition, the most stable reference genes for dsRNA treatment were used to evaluate the expression of target genes in insects treated with *RpII140* and *Prosα-2* dsRNA.

## Results

### Primer specificity and efficiency of candidate reference genes

PCR products generated by each primer pair using cDNA from *A. eugenii* as a template were visualized as single bands of the expected size on 1.5% agarose gel (Supplementary Fig. S1). The specificity of primer pairs was confirmed by sequencing of RT-PCR products and alignment with their corresponding gene fragment sequences. Additionally, the primer specificity was evaluated by melting curve analysis which showed the presence of a single peak (Supplementary Fig. S2). A standard curve was generated for each primer pair using a serial dilution of the cDNA in order to calculate the correlation coefficient (R^2^) and primer efficiency (E). E values varied from 93.65% to 108.39% and R^2^ values were superior to 0.993 (Table 1).

**Table 1:** Primers for the candidate reference genes and RNAi target genes used in the RT-qPCR analyses.

Gene	Acession number	Primer	Sequence 5′-3′	Size (bp)	Eff. (%)	R^2^
*β-ACT*	MH560343	β-ACT-Ae-qp-F	GGCATCCTCACCCTGAAATA	98	108.02	0.9938
		β-ACT-Ae-qp-R	CGCAGCTCGTTGTAGAAGGT			
*ArgK*	MK440119	ArgK-Ae-qp-F	CCCAGACAAAGTGGAGGAAA	113	103.92	0.9998
		ArgK-Ae-qp-R	TCTCCACTCGTGTCAGATGC			
*EF1-α*	MK440120	EF1-α-Ae-qp-F	TCTCCAAAAACGGACAGACC	100	98.42	0.9998
		EF1-α-Ae-qp-R	GGTTCAGTGGAATCCATTTTGT			
*GAPDH*	MH560346	GAPDH-Ae-qp-F	GACTTTACCGACAGCCTTGG	90	103.95	0.9976
		GAPDH-Ae-qp-R	CCCTCTGGAAAGTTGTGGAG			
*RPL12*	MK440124	RPL12-Ae-qp-F	TGTGATTTTCAGCCCTTTCC	80	101.66	0.9996
		RPL12-Ae-qp-R	GCCCTTTAGGTCTGTCACCA			
*RPS23*	MK440125	RPS23-Ae-qp-F	TTCCTACCGAAACCTGCAAC	97	102.25	0.9998
		RPS23-Ae-qp-R	AGAACGGCAAGAAAATCACG			
*α-TUB*	MK440121	α-TUB-Ae-qp-F	ACTGGTGTCCAACAGGTTTCA	93	105.73	0.9999
		α-TUB-Ae-qp-R	ACACGGCACGTTGTACCTTT			
*18S*	MK434327	18S-Ae-qp-F	CGCTAGCTGGCATCGTTTAT	117	91.95	0.9997
		18S-Ae-qp-R	ACGAACAGAAGCGAAAGCAT			
*28S*	MK434925	28S-Ae-qp-F	TGCCATCTCCCACTTATGCT	95	92.17	0.9956
		28S-Ae-qp-R	GGAAAAATTAGCGGGGAAAG			
*RpII140*	MK440123	RpII140-Ae-qp-F	ATAATCGAAGCGCACACTCC	108	96.52	0.9694
		RpII140-Ae-qp-R	CATGTCTCCCGATGATTTGA			
*Prosα-2*	MK440122	Prosα-2-Ae-qp-F	CGTTTTTGGAGAAAAGATACAGTG	86	105.63	0.9776
		Prosα-2-Ae-qp-R	CTCGAAGCTCTCCTTCAACG			

### Expression profile of candidate reference genes

**Figure 1:**
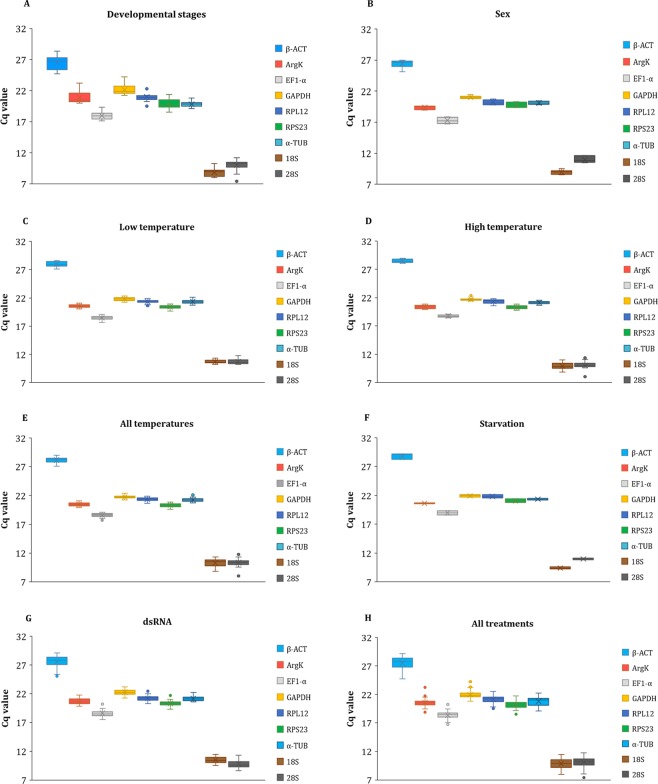
Expression profiles of candidate reference genes in different experimental conditions. Box and whisker plot chart showing the range of Cq values for each candidate reference gene under different treatments, developmental stages (**A**), sex (**B**), low temperature (**C**), high temperature (**D**), all temperatures (**E**), starvation (**F**), dsRNA (**G**) and in all treatments (**H**). The upper and lower edges of the boxes indicate the 75th and 25th percentiles, respectively. Whiskers represent the minimum and maximum Cq values, the line and the x within the box marks indicate the median and mean, respectively. Small circles indicate the outliers.

The mean quantification cycle (Cq) values varied considerably among the nine candidate reference genes, ranging from 8.82 (*18S*) in samples from different developmental stages to 28.83 (*β-ACT*) in samples from the starvation experiment (Fig. 1A,F). Overall, the candidate reference genes displayed similar expression patterns under different treatments. *18S* and *28S* had the lowest mean Cq values in all experimental conditions exhibiting the highest expression levels, whereas *β-ACT* and *GAPDH* showed the highest mean Cq values corresponding to the lowest expression levels (Fig. 1A–G). The mean Cq values of the reference genes considering all treatments varied from 9.86 (*18S*) to 27.45 (*GAPDH*) and the standard deviations (SD) of Cq values ranged from 0.58 (*RPS23*) to 1.16 (*β-ACT*) (Fig. 1H).

### Stability of candidate reference genes under different experimental conditions

#### Developmental stages

*EF1-α*, *18S*, *28S* and *α-TUB* were determined to be the most stable reference genes by BestKeeper and NormFinder among different developmental stages, while *RPL12*, *RPS23* and *EF1-α* were the top three most reliable reference genes according to geNorm. In contrast, geNorm and NormFinder ranked *β-ACT* and *ArgK* as the most unstable reference genes, whereas *ArgK* and *α-TUB* were considered the least appropriate genes by BestKeeper (Table 2).

**Table 2:** Stability of candidate reference genes according to BestKeeper, geNorm and NormFinder.

Experimental condition	Ranking	BestKeeper		geNorm		NormFinder	
		Gene	Coefficient correlation (r)	Gene	M value	Gene	Stability value
Developmental stages	1	*EF1-α*	0.930	*RPL12*	0.401	*EF1-α*	0.163
	2	*18S*	0.867	*RPS23*	0.414	*18S*	0.240
	3	*28S*	0.823	*EF1-α*	0.441	*α-TUB*	0.313
	4	*RPL12*	0.769	*28S*	0.539	*RPL12*	0.325
	5	*RPS23*	0.759	*18S*	0.584	*28S*	0.349
	6	*GAPDH*	0.606	*α-TUB*	0.624	*GAPDH*	0.401
	7	*β-ACT*	0.514	*GAPDH*	0.686	*RPS23*	0.411
	8	*α-TUB*	0.503	*ArgK*	0.737	*ArgK*	0.454
	9	*ArgK*	0.426	*β-ACT*	0.875	*β-ACT*	0.786
Sex	1	*RPS23*	0.969	*RPL12*	0.094	*α-TUB*	0.050
	2	*RPL12*	0.944	*RPS23*	0.095	*GAPDH*	0.079
	3	*GAPDH*	0.917	*EF1-α*	0.097	*RPS23*	0.134
	4	*28S*	0.916	*28S*	0.130	*RPL12*	0.156
	5	*EF1-α*	0.915	*α-TUB*	0.198	*18S*	0.183
	6	*α-TUB*	0.907	*GAPDH*	0.221	*EF1-α*	0.207
	7	*18S*	0.820	*18S*	0.236	*28S*	0.215
	8	*β-ACT*	0.206	*ArgK*	0.313	*ArgK*	0.298
	9	*ArgK*	−0.160	*β-ACT*	0.409	*β-ACT*	0.430
Low temperature	1	*GAPDH*	0.985	*GAPDH*	0.148	*GAPDH*	0.054
	2	*α-TUB*	0.933	*α-TUB*	0.156	*EF1A*	0.086
	3	*EF1-α*	0.930	*EF1-α*	0.159	*α-TUB*	0.107
	4	*18S*	0.912	*18S*	0.173	*18S*	0.110
	5	*28S/RPS23*	0.888	*ArgK*	0.190	*RPS23*	0.117
	6			*28S*	0.233	*ArgK*	0.119
	7	*ArgK*	0.832	*RPL12*	0.267	*28S*	0.157
	8	*β-ACT*	0.610	*β-ACT*	0.302	*RPL12*	0.178
	9	*RPL12*	0.578	*RPS23*	0.331	*β-ACT*	0.212
High temperature	1	*28S*	0.949	*α-TUB*	0.249	*GAPDH*	0.122
	2	*18S*	0.943	*RPS23*	0.251	*RPS23*	0.149
	3	*α-TUB*	0.922	*GAPDH*	0.261	*EF1-α*	0.152
	4	*RPS23*	0.837	*EF1-α*	0.276	*α-TUB*	0.174
	5	*GAPDH*	0.656	*RPL12*	0.296	*ArgK*	0.179
	6	*RPL12*	0.551	*β-ACT*	0.317	*β-ACT*	0.189
	7	*EF1-α*	0.207	*ArgK*	0.351	*RPL12*	0.251
	8	*ArgK*	0.076	*18S*	0.391	*28S*	0.307
	9	*β-ACT*	−0.152	*28S*	0.455	*18S*	0.735
All temperatures	1	*α-TUB*	0.917	*GAPDH*	0.235	*GAPDH*	0.101
	2	*28S*	0.907	*α-TUB*	0.256	*α-TUB*	0.111
	3	*18S*	0.866	*ArgK*	0.271	*RPS23*	0.122
	4	*RPS23*	0.816	*EF1-α*	0.315	*ArgK*	0.205
	5	*GAPDH*	0.807	*RPL12*	0.346	*EF1-α*	0.215
	6	*RPL12*	0.522	*RPS23*	0.369	*RPL12*	0.225
	7	*ArgK*	0.505	*β-ACT*	0.396	*18S*	0.307
	8	*EF1-α*	0.326	*18S*	0.446	*28S*	0.310
	9	*β-ACT*	−0.009	*28S*	0.490	*β-ACT*	0.360
Starvation	1	*RPL12*	1.006	*18S*	0.070	*RPL12*	0.020
	2	*RPS23*	1.001	*α-TUB*	0.080	*GAPDH*	0.036
	3	*α-TUB*	0.999	*RPL12*	0.090	*18S*	0.041
	4	*EF1-α*	0.988	*GAPDH*	0.100	*α-TUB*	0.058
	5	*18S*	0.979	*RPS23*	0.114	*RPS23*	0.070
	6	*ArgK*	0.946	*EF1-α*	0.124	*EF1-α*	0.090
	7	*GAPDH*	0.936	*ArgK*	0.143	*ArgK*	0.117
	8	*β-ACT*	0.829	*28S*	0.169	*28S*	0.190
	9	*28S*	0.281	*β-ACT*	0.220	*β-ACT*	0.270
dsRNA	1	*α-TUB*	0.839	*RPL12*	0.144	*α-TUB*	0.135
	2	*28S*	0.810	*RPS23*	0.147	*RPL12*	0.159
	3	*RPL12*	0.794	*α-TUB*	0.169	*RPS23*	0.188
	4	*RPS23*	0.793	*18S*	0.234	*GAPDH*	0.278
	5	*18S*	0.774	*28S*	0.251	*EF1-α*	0.279
	6	*EF1-α*	0.634	*EF1-α*	0.275	*18S*	0.311
	7	*ArgK*	0.485	*ArgK*	0.324	*ArgK*	0.325
	8	*GAPDH*	0.200	*GAPDH*	0.358	*28S*	0.388
	9	*β-ACT*	0.144	*β-ACT*	0.403	*β-ACT*	0.624

#### Sex

Different sets of suitable reference genes were identified by each algorithm when both sexes were evaluated. The most stable genes were *RPS23*, *RPL12* and *GAPDH* according to BestKeeper, *RPL12*, *RPS23* and *EF1-α* based on geNorm analysis, and *α-TUB*, *GAPDH* and *RPS23* by NormFinder. All algorithms indicated that *β-ACT* and *ArgK* exhibited the highest variations in expression (Table 2).

#### Low temperature

Consistent results were obtained by all algorithms which included *GAPDH*, *α-TUB* and *EF1-α* as the top three most stable reference genes under low temperature treatment. *β-ACT* and *RPL12* were ranked as the least stable genes by BestKeeper and NormFinder, and *β-ACT* and *RPS23* by geNorm (Table 2).

#### High temperature

*28S*, *18S* and *α-TUB* were ranked as the most suitable genes by BestKeeper, *α-TUB*, *RPS23* and *GAPDH* by geNorm, and *GAPDH*, *RPS23* and *EF1-α* by NormFinder under high temperature treatment. GeNorm and NormFinder identified *18S* and *28S* as the least stable genes, whereas BestKeeper indicated that *β-ACT* and *ArgK* had the worst performance (Table 2).

#### All temperatures

When the gene stability was evaluated in insects submitted to high and low temperature stresses, *GAPDH* and *α-TUB* were the most stably expressed reference genes followed by *ArgK* or *RPS23*, according to geNorm and NormFinder. Based on BestKeeper, *α-TUB*, *28S* and *18S* displayed the most stable expression. *β-ACT* and *EF1-α*; *28S* and *18S*; *β-ACT* and *28S* were the least stable genes according to BestKeeper, geNorm and NormFinder, respectively (Table 2).

#### Starvation

BestKeeper analysis revealed that *RPL12*, *RPS23* and *α-TUB* were the most stably expressed genes under starvation condition, while geNorm identified *18S*, *α-TUB* and *RPL12* as the most stable genes. According to results from NormFinder, *RPL12*, *GAPDH* and *18S* showed the highest stability. All algorithms indicated that *β-ACT* and *28S* were highly variable in their expression levels (Table 2).

#### dsRNA

Based on geNorm and NormFinder, *RPL12*, *RPS23* and *α-TUB* exhibited the highest expression stability for dsRNA exposure experiment, while BestKeeper indicated *α-TUB*, *28S* and *RPL12* as the most stable genes. BestKeeper and geNorm ranked *GAPDH* and *β-ACT* as the least stably expressed genes, while NormFinder indicated that *28S* and *β-ACT* were the least stable (Table 2).

### Overall ranking of candidate reference genes

**Figure 2:**
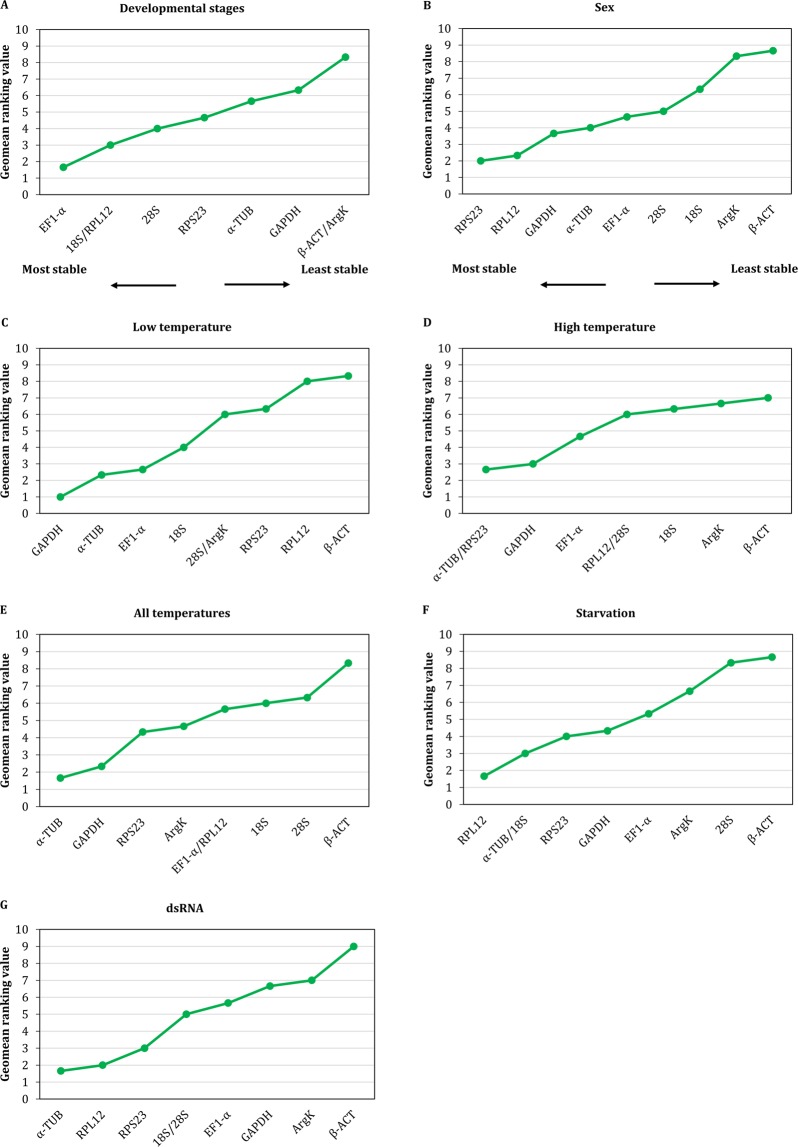
Comprehensive stability ranking of candidate reference genes based on BestKeeper, geNorm and NormFinder results.

As shown in Fig. 2 and Supplementary Table S1, the comprehensive ranking of candidate reference genes from the most to the least stable among the experimental conditions was as follows: *EF1-α* > *18S*/*RPL12* > *28S* > *RPS23* > *α-TUB* > *GAPDH* > *β-ACT*/*ArgK* across the developmental stages; *RPS23* > *RPL12* > *GAPDH* > *α-TUB* > *EF1-α* > *28S* > *18S* > *ArgK* > *β-ACT* for sex; *GAPDH* > *α-TUB* > *EF1-α* > *18S* > *28S*/*ArgK* > *RPS23* > *RPL12* > *β-ACT* for insects submitted to low temperatures; *α-TUB*/*RPS23* > *GAPDH* > *EF1-α* > *RPL12*/*28S* > *18S* > *ArgK* > *β-ACT* for insects exposed to high temperatures; *α-TUB* > *GAPDH* > *RPS23* > *ArgK* > *EF1-α*/*RPL12* > *18S* > *28S* > *β-ACT* when all temperatures where taken in account; *RPL12* > *α-TUB*/*18S* > *RPS23* > *GAPDH* > *EF1-α* > *ArgK* > *28S* > *β-ACT* for starvation stress; and *α-TUB* > *RPL12* > *RPS23* > *18S*/*28S* > *EF1-α* > *GAPDH* > *ArgK* > *β-ACT* for dsRNA treatment.

### Optimal number of candidate reference genes

**Figure 3:**
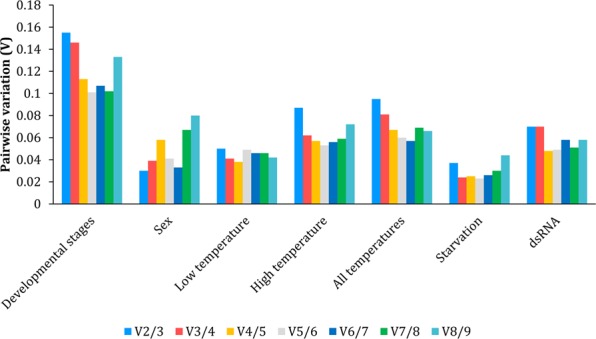
Determination of the optimal number of reference genes for accurate normalization using pairwise variation (Vn/n + 1) analysis by geNorm.

The pairwise variation (Vn/n + 1) analyses between two sequential normalization factors indicated that the pairwise variation V2/3 value was lower than the threshold value of 0.15 for sex, temperature, starvation and dsRNA treatments, suggesting that two reference genes are the optimal number of reference genes for accurate normalization of gene expression data in *A. eugenii* under these conditions. The pairwise variation V3/4 value was below the acceptable limit across developmental stages, therefore the use of three reference genes would be advisable to normalize the gene expression data (Fig. 3). The best combination of reference genes for normalization in each experimental condition is shown in Table 3.

**Table 3:** Recommended reference genes according to the experimental condition.

Experimental condition	Reference genes		
Developmental stages	*EF1-α*	*18S*	*RPL12*
Sex	*RPS23*	*RPL12*	
Low temperature	*GAPDH*	*α-TUB*	
High temperature	*α-TUB*	*RPS23*	
All temperatures	*α-TUB*	*GAPDH*	
Starvation	*RPL12*	*α-TUB*	
dsRNA	*α-TUB*	*RPL12*	

### Reference gene validation

**Figure 4:**
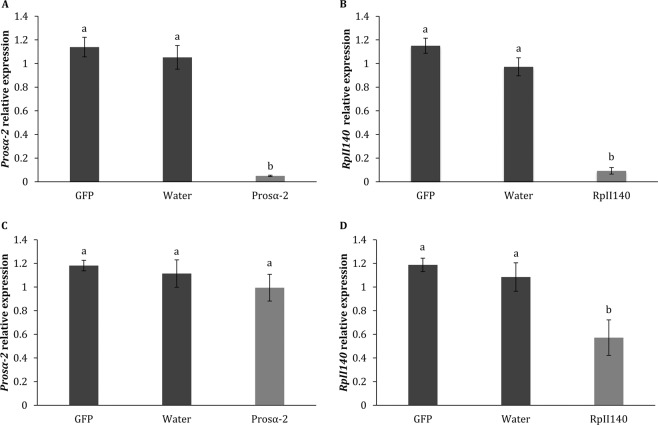
Relative expression of *Prosα-2* and *RpII140* genes using the recommended reference genes (*α-TUB* and *RPL12*) for normalization. Expression of target RNAi genes in insects injected with dsRNA (**A**,**B**) and insects fed on dsRNA (**C**,**D**). Data are expressed as mean ± standard deviation of error. Different letters indicate significant differences at P < 0.05 (One-way ANOVA followed by Tukey’s HSD test).

In order to validate some of the reference genes selected in this study, we evaluated the expression level of *DNA-directed RNA polymerase II subunit RPB2* (*RpII140*) and *Proteasome subunit alpha type2* (*Prosα-2*) genes in insects treated with the respective dsRNAs for these genes using the recommended set of reference genes (*α-TUB* and *RPL12*). We observed a significant gene knockdown of 92.2% and 96.5% in the insects injected with dsRNA targeting *RpII140* and *Prosα-2*, respectively, compared to the control treatment in which the insects were injected with *GFP* dsRNA. However, only the insects fed on *RpII140* dsRNA showed a significant decrease in gene expression level by 51.7% (Fig. 4).

## Discussion

RT-qPCR has been used extensively to assess gene expression in entomological research^17–20^. Normalization of RT-qPCR data with reference genes is one of the most common strategies used to correct experimental errors introduced through the steps of RT-qPCR analysis; however, the choice of reference genes with low expression variation is necessary to guarantee valid normalization and avoid inaccurate gene expression quantification^21,22^. Molecular studies provide important information about the genetic mechanisms underlying a variety of biological events and metabolic pathways, but only a few studies at the molecular level have been performed on *A. eugenii*. The selection of suitable reference genes for gene expression analysis will facilitate and boost such investigations in this important pest species.

In the present study, differences in the stability of potential reference genes were evaluated in order to select appropriate normalization factors for gene expression analysis in *A. eugenii*. Our results demonstrated that while some genes were ranked at the same position by BestKeeper, geNorm and NormFinder for a given condition, in general the stability ranking of the reference genes generated by these algorithms varied considerably. Variation in the ranking order has been observed in many studies and can be attributed to the different statistical approaches implemented in the algorithms^23,24^. To address this issue, a comprehensive ranking of reference genes was created based on the ranking value attributed by the algorithms geNorm, NormFinder and BestKeeper as performed previously^25–28^.

Our results indicated that *α-TUB* and *RPL12* were consistently ranked as the most stable genes according to the overall ranking and at least one of these genes was included in the set of normalizer genes suggested for each experimental condition. *α-TUB* is the main component of microtubules that form the cytoskeleton structure, which plays an important role in several eukaryotic cellular processes, including cell mobility and division, and intracellular trafficking^29^. Our results are consistent with previous studies reporting that *α-TUB* was stable for developmental stages, sexes, tissues, temperature and photoperiod stresses in *Empoasca onukii*^8^, among various tissues from *Coleomegilla maculata*^30^ and *Mythimna separata*^24^, for different temperatures in *Phenacoccus solenopsis*^31^ as well as for developmental stages, tissues and sexes in *Hermetia illucens*^32^. The *RPL12* gene encodes a structural protein of ribosomes and is involved in protein translation^33^. This gene exhibited high stability in *Acyrthosiphon pisum* exposed to temperature stresses^34^ and in *M. separata* under different photoperiod and temperature treatments, and larval tissues^35^.

In contrast to our results, *α-TUB* was not a suitable reference gene for normalization across developmental stages of *Cryptolestes ferrugineus*^36^, in different tissues of *Diaphorina citri*^37^, developmental stages, sexes and in response to temperature stress in *Propylea japonica*^38^, and in nonviruliferous/viruliferous *Frankliniella occidentalis*^39^. These results indicate that the gene stability can be affected by the biotic and abiotic conditions and even the insect species evaluated. As a consequence, the selection of condition-specific reference genes is strongly recommended prior RT-qPCR analysis.

Our analyses further identified *β-ACT* and *ArgK* as the least stable reference genes for most of the experimental conditions indicating that these genes are not ideal for RT-qPCR data normalization in *A. eugenii* under the conditions tested. Although *β-ACT* and *ArgK* are traditional reference genes and have been used in many gene expression studies, they are not always stably expressed. Consistent with our results, many studies have shown unstable expression of *β-ACT* under variable conditions in a variety of species, including *Bemisia tabaci*^40^, *M. separata*^35^, *F. occidentalis*^39^, *Henosepilachna vigintioctopunctata*^41^, *P. japonica*^38^, *Hippodamia convergens*^42^ and *C. maculata*^30^, as well as for *ArgK* in *Spodoptera litura*^43^, *P. japonica*^38^, *C. maculata*^30^ and *E. onukii*^8^.

The evaluation of gene expression is a fundamental and routine analysis in RNAi-related studies, and therefore the choice of adequate reference genes is crucial to achieve precise results. We demonstrated that the selected reference genes were suitable to measure the relative expression of the target genes *Prosα-2* and *RpII140* in insects treated with dsRNA corresponding to these genes. The reference genes *α-TUB* and *RPL12* identified in our work can be useful for normalization of target gene expression levels in further research on RNAi in *A. eugenii*. To date, neither the genomic or transcriptomic data are available for *A. eugenii*; however, this sequencing information could provide a valuable resource to gain a deeper understanding of different molecular mechanisms and to discover potential target genes that can be used in RNAi-mediated control methods against this insect pest. Future studies should focus on genome and transcriptome sequencing to fill the gap of genetic information on *A. eugenii*.

Despite the importance of accessing reference gene stability, these analyses are often not performed prior gene expression studies^44,45^. Our results highlighted the need to adopt this practice for proper normalization of RT-qPCR data. We found that the best combinations of reference genes that should be used as internal controls were *EF1-α*, *18S* and *RPL12* for developmental stages; *RPS23* and *RPL12* for sex, *GAPDH* and *α-TUB* for low temperature; *α-TUB* and *RPS23* for high temperature; *α-TUB* and *GAPDH* for all temperatures; *RPL12* and *α-TUB* for starvation; *α-TUB* and *RPL12* for dsRNA treatment. The selected reference genes may be helpful in further gene expression studies in *A. eugenii*. In addition, the set of primers validated in this study can be used to evaluate the suitability of the candidate reference genes in experimental conditions other than those tested here.

## Methods

### Insect colony

*A. eugenii* colony was maintained at 27 ± 10 °C, 30 ± 5% relative humidity, 14:10 h light:dark photoperiod and supplied with jalapeno peppers (*Capsicum annuum*) as an oviposition substrate and food source for both larvae and adults.

### Reference gene selection, gene fragment cloning and primer design

Based on a literature search, we selected nine genes (*β-ACT*, *ArgK*, *EF1-α*, *GAPDH*, *RPL12*, *RPS23*, α-*TUB*, *18S* and *28S*) commonly used as reference genes and that have shown high stability in other insect species to investigate their suitability as reference genes for RT-qPCR in *A. eugenii*^7,8,30,34,43,46^. Degenerate primers for *RPS23*, *18S*, *28S* and *RpII140* were manually designed based on conserved nucleotide sequence among other Coleoptera species. Primers for *ArgK*, *EF1-α*, *RPL12*, α-*TUB* and *Prosα-2* were obtained from previous works^30,47^ (Supplementary Table S2). PCR amplifications consisted of 5 µL of 10x PCR buffer, 8 µL of MgCl_2_ (50 mM), 5 µL of dNTPs mix (10 mM of each nucleotide), 8 µL of each primer (10 µM), 1.25 µL of Taq DNA Polymerase (5U/µL) (ThermoFisher Scientific), 2 µL of cDNA (diluted 10×) and 12.75 µL of nuclease-free water. The PCR cycling conditions were as follows: one cycle of 95 °C for 3 min; 40 cycles of 95 °C for 30 sec, 50–55 °C for 30 sec and 72 °C for 30 sec; a final cycle of 72 °C for 5 min. PCR products were purified using QIAquick Gel Extraction Kit (Qiagen) and cloned into the pJET1.2/blunt cloning vector using the CloneJET PCR Cloning Kit (Thermo Scientific) following the manufacturer’s protocol. Recombinant plasmids were transformed into One Shot^®^ TOP10 Chemically Competent *Escherichia coli* cells (Invitrogen) and sequenced by GENEWIZ company (South Plainfield, NJ, USA). According to the partial sequences of the genes, specific primers were designed using Primer3Plus software^48^ (http://www.bioinformatics.nl/cgi-bin/primer3plus/primer3plus.cgi). Primers for *β-ACT* gene were designed based on the sequence available on NCBI (Accession number MH560343) and the primers for *GAPDH* were obtained from a previous work^49^ (Table 1).

### Experimental treatments

#### Developmental stages

Samples of each developmental stage of *A. eugenii* including eggs, first, second and third instars, pupae and adults were collected. Each biological replicate included 20 eggs, 18 first instar larvae, 12 second instar larvae, 10 third instar larvae, 6 pupae or 6 adults (3 females and 3 males).

#### Sex

Six adults of *A. eugenii* (males or females) were pooled as one biological replicate.

#### Starvation

*A. eugenii* adults were placed in a plastic vial and starved for 24 h at 27 ± 1 °C, 75 ± 5% relative humidity in a growth chamber. Each biological replicate consisted of six insects.

#### Temperature

To examine the effects of temperature on gene expression stability, *A. eugenii* adults were exposed to temperatures ranging from 5–40 °C at 5 °C increments for 3 h in a climate-controlled chamber. Insects maintained at 5–20 °C were included in the low temperature treatment, while insects maintained at 30–40 °C were included in the high temperature treatment. Insects exposed to 25 °C constituted the control group for both treatments. Each biological replicate consisted of six adults. Gene expression stability was also evaluated when insects exposed to all temperatures were taken into account.

#### dsRNA

For the RNAi experiments, we selected two target genes (*RpII140* and *Prosα-2*) that could potentially be used in RNAi-mediated control methods against *A. eugenii* based on previous studies demonstrating mortality of insects exposed to dsRNA targeting homologs of these genes^50,51^. DNA template for the synthesis of dsRNA was amplified by PCR using gene-specific primers containing a T7 polymerase promoter sequence at the 5′ (Table 4) from plasmid DNA. The PCR reaction was purified using QIAquick Gel Extraction Kit (Qiagen) and the dsRNA synthesized and purified using the MEGAScript^TM^ RNAi Kit (Invitrogen) according to the manufacturer’s instructions. For the bioassay by microinjection, *A. eugenii* adults (11–12 insects) were injected dorsally with 0.5 µL of *RpII140* dsRNA or *Prosα-2* dsRNA at 1000 ng/µL using the IM-11–2 microinjector (Narishige). Control insects were injected with an equivalent amount of *GFP* dsRNA or nuclease-free water. After the injections, insects were maintained at 27 ± 1 °C, 75 ± 5% relative humidity and fed on pepper. Biological replicates of each treatment were collected three days post-injection. Each biological replicate consisted of one insect. In the feeding bioassay, a droplet consisting of 24 µL of 20% sucrose solution with green food dye containing *RpII140* dsRNA or *Prosα-2* dsRNA at a concentration of 500 ng/µL was offered to twelve *A. eugenii* adults placed in a plastic vial. *GFP* dsRNA and nuclease-free water were used as controls. The droplets were replaced on the third day. Biological replicates consisting of a pool of three insects were collected on the fifth day.

**Table 4:** Primers used for dsRNA synthesis.

Primer	Sequence 5′−3′	Size (bp)
RpII140-Ae-ds-F	**TAATACGACTCACTATAGGG**AGCGGGATGAATCTCACAGT	334
RpII140-Ae-ds-R	**TAATACGACTCACTATAGGG**GCGTCAGATGGACATTATCG	
Prosα-2-Ae-ds-F	**TAATACGACTCACTATAGGG**CGCAACGGAAAATAAACACA	338
Prosα-2-Ae-ds-R	**TAATACGACTCACTATAGGG**TCCATGCAAAGTAAGCTCCA	

Bold letters represent the T7 promoter sequence.

The collected samples from all experimental treatments performed in this study were placed in centrifuge tubes, rapidly flash-frozen in liquid nitrogen, and stored at -80 °C until RNA extraction. Each treatment included three biological replicates.

### RNA extraction and cDNA synthesis

Total RNA was extracted using the RNeasy Mini Kit (Qiagen) and on-column genomic DNA digestion was performed using the RNase-free DNase Set (Qiagen) as recommended by the manufacturer. RNA samples were quantified by Nanodrop 1000 spectrophotometer (Thermo Scientific). The absence of DNA contamination and the RNA integrity was analyzed on a 1.5% agarose gel. Total RNA (500 ng) was reverse transcribed to cDNA using a High-Capacity cDNA Reverse Transcription Kit (Applied Biosystems) following the manufacturer’s protocol. The cDNA was diluted 50-fold with nuclease-free water for subsequent RT-qPCR assays.

### Reverse-transcription quantitative PCR (RT-qPCR)

The RT-qPCR assays were carried out using a BioRad CFX96 qPCR System (Bio-Rad) with an optical 96-well plate. Each RT-qPCR reaction mix contained 2 μL of cDNA diluted 50×, 5 μL of SsoAdvanced^TM^ Universal SYBR^®^ Green Supermix (Bio-Rad), 0.2 μL of each primer at 10 μM and 3.6 μL of nuclease-free water for a total volume of 10 μL. Thermal cycling conditions were 95 °C for 2 min, followed by 40 cycles of 95 °C for 5 sec and 60 °C for 30 sec. To assure the specificity of the primers and to eliminate the possibility of primer dimer formation, melting curves ranging from 65 °C to 95 °C with 0.5 °C/5 sec increment were included after amplification. Non-template control (NTC) was used as a negative control for each master mix. Assays were performed with three biological replicates each comprising three technical replicates. The PCR amplification efficiency of the primer pairs was determined from the standard curve generated with 5-fold serial dilutions of cDNA.

### Analysis of the stabilities of candidate reference genes

Three algorithms, geNorm accessed as part of the qbase+ analysis software from Biogazelle^52^ (http://medgen.ugent.be/*jvdesomp/genorm/), NormFinder version 0.953^53^ (https://www.moma.dk/normfinder-software) and BestKeeper version 1^54^ (https://www.gene-quantification.de/bestkeeper.html) were used to evaluate the expression stability of the candidate reference genes. For NormFinder analyses, Cq values were transformed into non-normalized relative quantities according to the formula: (E)^ΔCq^ where E represents the primer efficiency for each gene and ΔCq represents the lowest Cq value - Cq value of each sample. Raw Cq values were employed in geNorm and BestKeeper analyses. The comprehensive ranking of the reference genes was based on the geometric mean of geNorm, NormFinder, and BestKeeper results.

BestKeeper calculates the standard deviation (SD) and coefficient of variation (CV) based on the Cq values for each candidate reference gene. Genes with SD greater than 1 are considered unstable. It also estimates the BestKeeper Index and then calculates the correlation coefficient (r) between each candidate gene and the BestKeeper index. Genes with higher stability have higher r values. GeNorm evaluates the stability of the potential reference gene based on an “M” value which represents the average pairwise variation of a specific candidate reference gene with all other genes. The genes with the lowest M values are considered the most stably expressed. For homogeneous samples, suitable reference genes should have M values lower than 0.5, but if the samples are considered heterogeneous M values up to 1 are acceptable. GeNorm was used to determine the optimal number of reference genes required for accurate normalization. It calculates the pairwise variation values (Vn/n + 1) between two consecutively ranked normalization factors (NFn and NFn+1), where n is the number of reference genes used in the normalization factor. The stepwise inclusion of the subsequent more stable reference gene can result in an increase or decrease in Vn/n + 1 value and a Vn/n + 1 below 0.15 indicates that the inclusion of an additional reference gene is not necessary for normalization. NormFinder analyzes the intra- and inter-group variations to estimate the stability of each candidate reference gene. Lower stability values indicate higher gene expression stability.

### Validation of the reference genes in *A. eugenii*

The combination of the most stable reference genes (*α-TUB* and *RPL12*) in insects treated with dsRNA was validated by evaluating the expression of *Prosα-2* and *RpII140* genes. The relative expression of the target genes was calculated using the 2^−^ ^ΔΔCt^ method^55^. Three technical and three biological replicates were performed in this analysis. Gene expression data were analyzed by one-way ANOVA followed by Tukey’s HSD test at P < 0.05. All statistical analyses were performed using JMP Pro 13 Software (SAS Institute, Cary, NC). Data are presented as mean ± standard error of the mean (SE).

## Supplementary information

Selection of reference genes for normalization of RT-qPCR data in gene expression studies in Anthonomus eugenii Cano (Coleoptera: Curculionidae).

## Data Availability

All relevant data analyzed during this study are included in this article and its Supplementary Information files.

## References

[1] Wong ML, Medrano JF (2005). Real-time PCR for mRNA quantitation. Biotechniques.

[2] Schmittgen TD, Livak KJ (2008). Analyzing real-time PCR data by the comparative Ct method. Nat. Protoc..

[3] Bustin SA (2009). The MIQE Guidelines: Minimum Information for Publication of Quantitative Real-Time PCR Experiments. Clin. Chem.

[4] Kozera B, Rapacz M (2013). Reference genes in real-time PCR. J. Appl. Genet.

[5] García-Reina A, Rodríguez-García MJ, Galián J (2018). Validation of reference genes for quantitative real-time PCR in tiger beetles across sexes, body parts, sexual maturity and immune challenge. Sci. Rep.

[6] Sagri E (2017). Housekeeping in Tephritid insects: the best gene choice for expression analyses in the medfly and the olive fly. Sci. Rep.

[7] Yang X, Pan H, Yuan L, Zhou X (2018). Reference gene selection for RT-qPCR analysis in *Harmonia axyridis*, a global invasive lady beetle. Sci. Rep..

[8] Yu Y (2018). Reference genes selection for quantitative gene expression studies in tea green leafhoppers, *Empoasca onukii* Matsuda. PLoS One.

[9] Basu S (2019). Evaluation of reference genes for real-time quantitative PCR analysis in southern corn rootworm, *Diabrotica undecimpunctata* howardi (Barber). Sci. Rep..

[10] O’Brien CW, Wibmer GJ (1982). Annotated checklist of the weevils (Curculionidae sensu lato) of North America, Central America, and the West Indies (Coleoptera: Curculionoidea). Mem. Am. Entomol. Institute, Ann. Arbor, MI.

[11] Burke HR, Woodruff RE (1980). The pepper weevil (*Anthonomus eugenii* Cano) in Florida (Coleoptera: Curculionidae). Fla. Dept. Agr. Consum. Serv. Entomol. Cir..

[12] Andrews KL (1986). A supervised control programme for the pepper weevil, *Anthonomus eugenii* Cano, in Honduras, Central America. Trop. Pest Manag..

[13] Elmore JC, Davis C, Campbell RE (1934). The pepper weevil. USDA Tech. Bull. n.

[14] Schultz, P. B. & Kuhar, T. P. First record of pepper weevil infestation in Virginia. *Plant Health Progress*, 10.1094/php-2008-0118-01-br (2008).

[15] Ingerson-Mahar J, Eichinger B, Holmstrom K (2015). How does pepper weevil (Coleoptera: Curculionidae) become an important pepper pest in New Jersey?. J. Integr. Pest Manag.

[16] Seal D, Martin C, Seal DR, Martin CG (2016). Pepper weevil (Coleoptera: Curculionidae) preferences for specific pepper cultivars, plant parts, fruit colors, fruit sizes, and timing. Insects.

[17] Wang J, Zhang RR, Gao GQ, Ma MY, Chen H (2016). Cold tolerance and silencing of three cold-tolerance genes of overwintering Chinese white pine larvae. Sci. Rep.

[18] Meagher R (2017). Mechanism and DNA-based detection of field-evolved resistance to transgenic Bt corn in fall armyworm (*Spodoptera frugiperda*). Sci. Rep..

[19] Mello TRP (2014). Developmental regulation of ecdysone receptor (EcR) and EcR-controlled gene expression during pharate-adult development of honeybees (*Apis mellifera*). Front. Genet..

[20] Wang Z, Zhou W, Hameed MS, Liu J, Zeng X (2018). Characterization and expression profiling of neuropeptides and g-protein-coupled receptors (GPCRs) for neuropeptides in the asian citrus psyllid, *Diaphorina citri* (Hemiptera: Psyllidae). Int. J. Mol. Sci..

[21] Guénin S (2009). Normalization of qRT-PCR data: The necessity of adopting a systematic, experimental conditions-specific, validation of references. J. Exp. Bot..

[22] Huggett J, Dheda K, Bustin S, Zumla A (2005). Real-time RT-PCR normalisation; strategies and considerations. Genes Immun..

[23] Zhang Y (2017). Selection and validation of appropriate reference genes for quantitative real-time PCR normalization in staminate and perfect flowers of andromonoecious *Taihangia rupestris*. Front. Plant Sci..

[24] Li H-B (2018). Screening potential reference genes for quantitative real-time PCR analysis in the oriental armyworm, *Mythimna separata*. PLoS One.

[25] Pombo MA (2019). Transcriptome-based identification and validation of reference genes for plant-bacteria interaction studies using *Nicotiana benthamiana*. Sci. Rep..

[26] Lacerda ALM (2015). Reference gene selection for qPCR analysis in tomato-bipartite begomovirus interaction and validation in additional tomato-virus pathosystems. PLoS One.

[27] Gong H (2016). Evaluation of candidate reference genes for RT-qPCR studies in three metabolism related tissues of mice after caloric restriction. Sci. Rep.

[28] Hossain MS, Ahmed R, Haque MS, Alam MM, Islam MS (2019). Identification and validation of reference genes for real-time quantitative RT-PCR analysis in jute. BMC Mol. Biol..

[29] Nielsen MG, Gadagkar SR, Gutzwiller L (2010). Tubulin evolution in insects: gene duplication and subfunctionalization provide specialized isoforms in a functionally constrained gene family. BMC Evol. Biol..

[30] Yang C (2016). Selection of reference genes for RT-qPCR analysis in a predatory biological control agent, *Coleomegilla maculata* (Coleoptera: Coccinellidae). Sci. Rep..

[31] Arya SK (2017). Reference genes validation in *Phenacoccus solenopsis* under various biotic and abiotic stress conditions. Sci. Rep..

[32] Gao Z, Deng W, Zhu F (2019). Reference gene selection for quantitative gene expression analysis in black soldier fly (*Hermetia illucens*). PLoS One.

[33] Nagaraj S, Senthil-Kumar M, Ramu VS, Wang K, Mysore KS (2016). Plant ribosomal proteins, RPL12 and RPL19, play a role in nonhost disease resistance against bacterial pathogens. Front. Plant Sci..

[34] Yang C, Pan H, Liu Y, Zhou X (2014). Selection of reference genes for expression analysis using quantitative real-time PCR in the pea aphid, *Acyrthosiphon pisum* (Harris) (Hemiptera, Aphidiae). PLoS One.

[35] Li K, Xu N, Yang YJ, Zhang JH, Yin H (2018). Identification and validation of reference genes for RT-qPCR normalization in *Mythimna separata* (Lepidoptera: Noctuidae). Biomed Res. Int..

[36] Tang PA, Duan JY, Wu HJ, Ju XR, Yuan ML (2017). Reference gene selection to determine differences in mitochondrial gene expressions in phosphine-susceptible and phosphine-resistant strains of *Cryptolestes ferrugineus*, using qRT-PCR. Sci. Rep..

[37] Bin S (2018). Selection of reference genes for optimal normalization of quantitative real-time polymerase chain reaction results for *Diaphorina citri* adults. J. Econ. Entomol..

[38] Lü J (2018). Selection of appropriate reference genes for RT-qPCR analysis in *Propylea japonica* (Coleoptera: Coccinellidae). PLoS One.

[39] Yang C (2015). Stable reference gene selection for RT-qPCR analysis in nonviruliferous and viruliferous *Frankliniella occidentalis*. PLoS One.

[40] Dai T-M, Lü Z-C, Liu W-X, Wan F-H (2017). Selection and validation of reference genes for qRT-PCR analysis during biological invasions: The thermal adaptability of *Bemisia tabaci* MED. PLoS One.

[41] Lü J (2018). Selection and validation of reference genes for RT-qPCR analysis of the ladybird beetle *Henosepilachna vigintioctopunctata*. Front. Physiol..

[42] Pan H, Yang X, Siegfried BD, Zhou X (2015). A comprehensive selection of reference genes for RT-qPCR analysis in a predatory lady beetle, *Hippodamia convergens* (Coleoptera: Coccinellidae). PLoS One.

[43] Lu Y (2013). Identification and validation of reference genes for gene expression analysis using quantitative PCR in *Spodoptera litura* (Lepidoptera: Noctuidae). PLoS One.

[44] Busato S, Mezzetti M, Logan P, Aguilera N, Bionaz M (2019). What’s the norm in normalization? A frightening note on the use of RT-qPCR in the livestock science. Gene X.

[45] Chapman JR, Waldenström J (2015). With reference to reference genes: A systematic review of endogenous controls in gene expression studies. PLoS One.

[46] Fu W (2013). Exploring valid reference genes for quantitative real-time PCR analysis in *Plutella xylostella* (Lepidoptera: Plutellidae). Int. J. Biol. Sci..

[47] Pinheiro DH, Taylor CE, Wu K, Siegfried BD (2019). Delivery of gene-specific dsRNA by microinjection and feeding induces RNAi response in Sri Lanka weevil, *Myllocerus undecimpustulatus undatus* Marshall. Pest Manag. Sci..

[48] Untergasser A (2007). Primer3Plus, an enhanced web interface to Primer3. Nucleic Acids Res.

[49] Wu K (2019). Lethal RNA interference response in the pepper weevil. J. Appl. Entomol..

[50] Christiaens O (2016). RNA interference: A promising biopesticide strategy against the African Sweetpotato Weevil *Cylas brunneus*. Sci. Rep..

[51] Knorr E (2018). Gene silencing in *Tribolium castaneum* as a tool for the targeted identification of candidate RNAi targets in crop pests. Sci. Rep..

[52] Vandesompele J (2002). Accurate normalization of real-time quantitative RT-PCR data by geometric averaging of multiple internal control genes. Genome Biol..

[53] Andersen CL, Jensen JL, Ørntoft TF (2004). Normalization of real-time quantitative reverse transcription-PCR data: A model-based variance estimation approach to identify genes suited for normalization, applied to bladder and colon cancer data sets. Cancer Res..

[54] Pfaffl MW, Tichopad A, Prgomet C, Neuvians TP (2004). Determination of stable housekeeping genes, differentially regulated target genes and sample integrity: BestKeeper – Excel-based tool using pair-wise correlations. Biotechnol. Lett..

[55] Livak KJ, Schmittgen TD (2001). Analysis of relative gene expression data using real-time quantitative PCR and the 2^−ΔΔCT^ method. Methods.

