# Supplementary material for: Selection of reference genes for normalization of RT-qPCR data in gene expression studies in Anthonomus eugenii Cano (Coleoptera: Curculionidae)
Source: Sci Rep. 2020 Mar 19;10:5070. doi: 10.1038/s41598-020-61739-z (PMC7081190; doi:10.1038/s41598-020-61739-z)
Supplement: Supplementary file 1 — Selection of reference genes for normalization of RT-qPCR data in gene expression studies in Anthonomus eugenii Cano (Coleoptera: Curculionidae). [file 41598_2020_61739_MOESM1_ESM.pdf]

Supplementary information

Selection of reference genes for normalization of RT-qPCR data in gene expression studies in *Anthonomus eugenii* Cano (Coleoptera: Curculionidae)

Daniele H. Pinheiro [1, 2] & Blair D. Siegfried [1*]

[1] University of Florida, Entomology and Nematology Department, Charles Steinmetz Hall, P. O. Box 110620, Gainesville, FL, 32611–0620, United States.

[2] Present address: Embrapa Genetic Resources and Biotechnology, Parque Estação Biológica, W5 Norte, P. O. Box 02372, Brasília, DF, 70770–917, Brazil.

* Correspondence and requests for materials should be addressed to B.D.S. (email: bsiegfried1@ufl.edu).

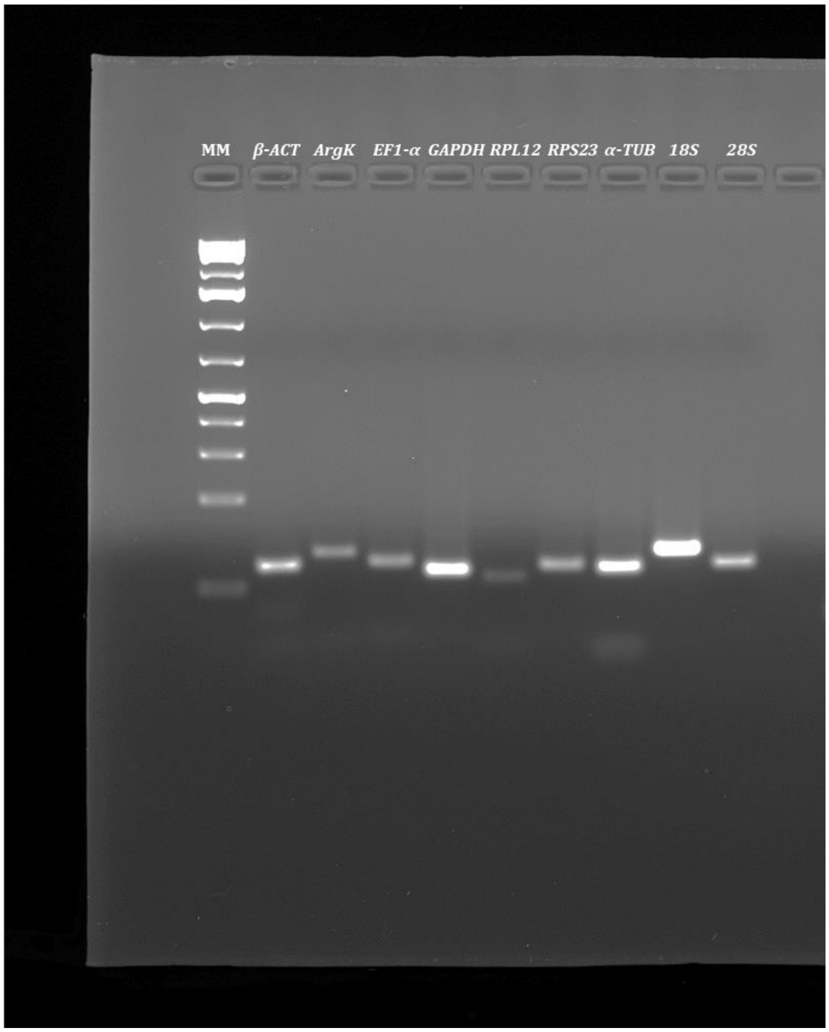

Supplementary Fig. S1. Agarose gel (1.5%) indicates the amplification of a single fragment of candidate reference genes.

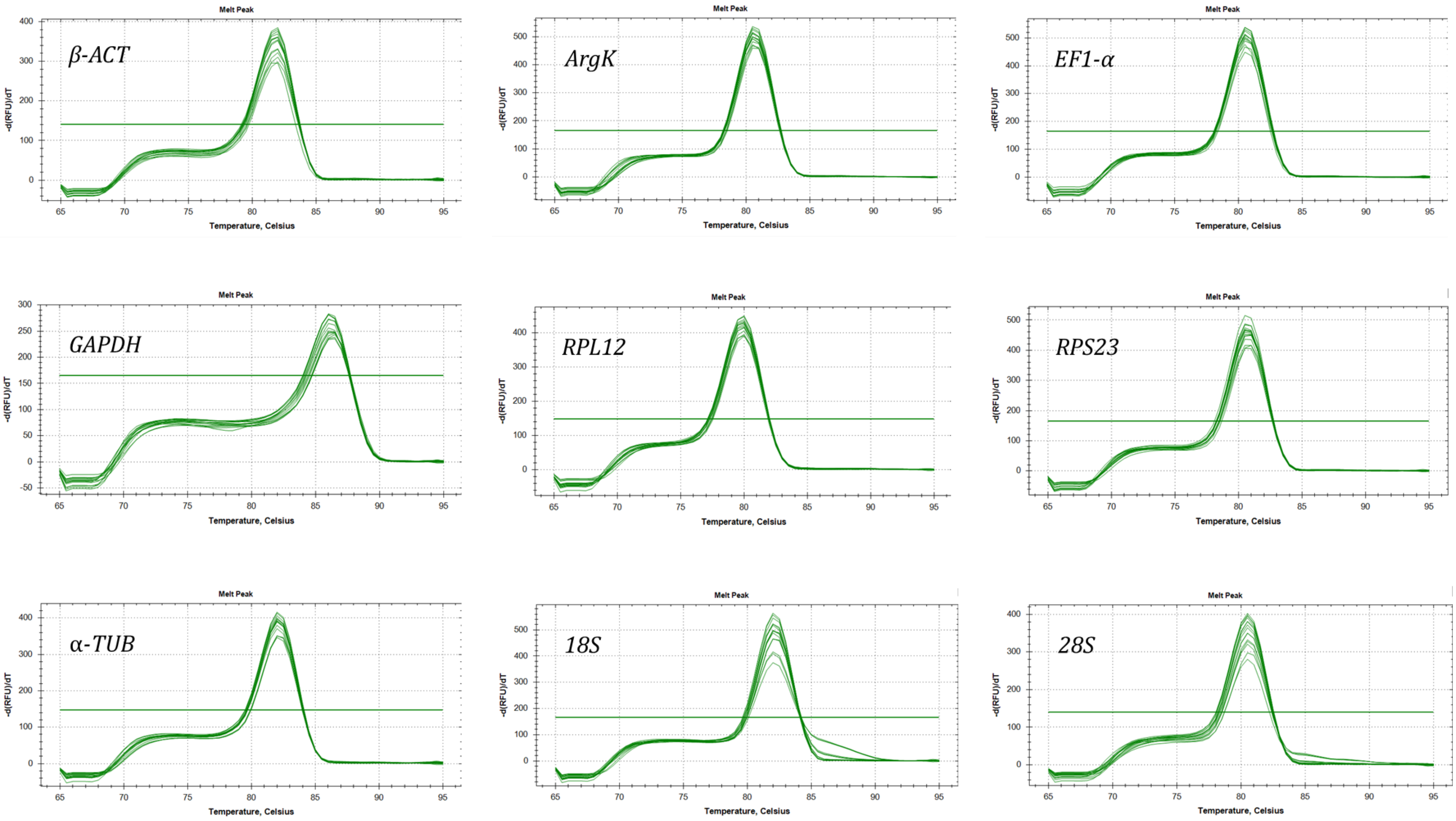

Supplementary Fig. S2. Melting curves generated by RT-qPCR.

Table S1. Comprehensive stability ranking of candidate reference genes according to BestKeeper, geNorm and NormFinder.

Experimental condition	Comprehensive ranking	Gene	BestKeeper	GeNorm	NormFinder	Mean
Developmental stages	1	*EF1-α*	1	3	1	1.66
	2	*18S*	2	5	2	3
	2	*RPL12*	4	1	4	3
	3	*28S*	3	4	5	4
	4	*RPS23*	5	2	7	4.66
	5	*α-TUB*	8	6	3	5.66
	6	*GAPDH*	6	7	6	6.33
	7	*β-ACT*	7	9	9	8.33
	7	*ArgK*	9	8	8	8.33
Sex	1	*RPS23*	1	2	3	2
	2	*RPL12*	2	1	4	2.33
	3	*GAPDH*	3	6	2	3.66
	4	*α-TUB*	6	5	1	4
	5	*EF1-α*	5	3	6	4.66
	6	*28S*	4	4	7	5
	7	*18S*	7	7	5	6.33
	8	*ArgK*	9	8	8	8.33
	9	*β-ACT*	8	9	9	8.66

Continued

Low temperature	1	*GAPDH*	1	1	1	1
	2	*α-TUB*	2	2	3	2.33
	3	*EF1-α*	3	3	2	2.66
	4	*18S*	4	4	4	4
	5	*28S*	5	6	7	6
	5	*ArgK*	7	5	6	6
	6	*RPS23*	5	9	5	6.33
	7	*RPL12*	9	7	8	8
	8	*β-ACT*	8	8	9	8.33
High temperature	1	*α-TUB*	3	1	4	2.66
	1	*RPS23*	4	2	2	2.66
	2	*GAPDH*	5	3	1	3
	3	*EF1-α*	7	4	3	4.66
	4	*RPL12*	6	5	7	6
	4	*28S*	1	9	8	6
	5	*18S*	2	8	9	6.33
	6	*ArgK*	8	7	5	6.66
	7	*β-ACT*	9	6	6	7
All temperatures	1	*α-TUB*	1	2	2	1.66
	2	*GAPDH*	5	1	1	2.33
	3	*RPS23*	4	6	3	4.33
	4	*ArgK*	7	3	4	4.66
	5	*EF1-α*	8	4	5	5.66
	5	*RPL12*	6	5	6	5.66
	6	*18S*	3	8	7	6
	7	*28S*	2	9	8	6.33
	8	*β-ACT*	9	7	9	8.33

Continued

Starvation	1	*RPL12*	1	3	1	1.66
	2	*α-TUB*	3	2	4	3
	2	*18S*	5	1	3	3
	3	*RPS23*	2	5	5	4
	4	*GAPDH*	7	4	2	4.33
	5	*EF1-α*	4	6	6	5.33
	6	*ArgK*	6	7	7	6.66
	7	*28S*	9	8	8	8.33
	8	*β-ACT*	8	9	9	8.66
dsRNA	1	*α-TUB*	1	3	1	1.66
	2	*RPL12*	3	1	2	2
	3	*RPS23*	4	2	3	3
	4	*18S*	5	4	6	5
	4	*28S*	2	5	8	5
	5	*EF1-α*	6	6	5	5.66
	6	*GAPDH*	8	8	4	6.66
	7	*ArgK*	7	7	7	7
	8	*β-ACT*	9	9	9	9

Table S2. Sequence and amplicon size of the primers used for cloning.

Primer	Sequence 5'-3'	Tm (°C)	Size (bp)	Reference
ArgK-F	CCTGTTCGACCCTATCATCGARGAYTAYCA	55	733	Yang et al., 2016
ArgK-R	GTCGTAGATACCACCTTCAGCTTCNGTRTGYTC			
EF1-α-F	GCCGGTACYGGWGAATTCGAAGC	55	290	Pinheiro et al., 2019
EF1-α-R	CATCCCTTGAACCABGGCATCTT			
RPL12-F	ATGCCMCCAAARTTCGAYCCSA	55	365	Pinheiro et al., 2019
RPL12-R	GCCATDGAKCKDGGCCTCATTTG			
RPS23-F	TGGGCYGACAARGAKTACAARAAAGC	55	300	This study
RPS23-R	CCTTRAATCTDACWCCRGGAATATC			
α-TUB-F	ATCAAGACCAARCGTACCATCC	55	257	Pinheiro et al., 2019
α-TUB-R	TCAGAGAAYTCWCCTTCYTCCATACC			
18S-F	GTTGCGGTTAAAAAGCTCGT	50	644	This study
18S-R	GTGAGGTTTCCCGTGTTGAG			
28S-F	CGAGATTCCCACTGTCCCTA	55	532	This study
28S-R	ACTGAACATCGGGATCAAGC			
RpII140-F	TTGGAGTTYTTGGAGGAGTGGTC	55	581	This study
RpII140-R	CCCATGGCTTGYTTDCCCAT			
Prosα-2-F	CAAATYGARTATGCCTTRGCTGC	50	581	Pinheiro et al., 2019
Prosα-2-R	TCACAAATHCCDACTTCAATRTTATC			

Tm - Melting temperature used in PCR amplification reactions.

References

Yang, C. *et al.* Selection of reference genes for RT-qPCR analysis in a predatory biological control agent, *Coleomegilla maculata* (Coleoptera: Coccinellidae). *Sci. Rep.* **5**, 18201 (2016).

Pinheiro, D. H., Taylor, C. E., Wu, K. & Siegfried, B. D. Delivery of gene-specific dsRNA by microinjection and feeding induces RNAi response in Sri Lanka weevil, *Myllocerus undecimpustulatus undatus* Marshall. *Pest Manag. Sci.* (2019). doi:10.1002/ps.5601
